# Social Relationships, Depressive Symptoms, and Self-Rated Health Among Adults Living Alone in South Korea: An Analysis of the 2025 Korea Community Health Survey

**DOI:** 10.3390/healthcare14213793

**Published:** 2026-11-06

**Authors:** Man Soo Kim

**Affiliations:** Korea Counseling Psychology Support Center, Dongducheon 11343, Republic of Korea; symsy@hanmail.net

**Keywords:** living alone, social relationships, depressive symptoms, self-rated health, neighborhood trust, Korea Community Health Survey

## Abstract

Background/Objectives: Social relationships may be associated with psychological and perceived general health among adults living alone. This study examined the associations of contact frequency with family members or relatives, neighbors, and friends, and perceived neighborhood trust with depressive symptoms and self-rated health among adults living alone in South Korea, including potential age-related differences. Methods: Data from 44,168 adults living alone who participated in the 2025 Korea Community Health Survey were analyzed. Complex-samples regression models accounted for stratification, clustering, and sampling weights. Clinically relevant depressive symptoms were defined as a Patient Health Questionnaire-9 score of 10 or higher. Poor self-rated health was assessed separately. Multivariable models adjusted for demographic, socioeconomic, and selected health-related characteristics. Age-related interactions and sensitivity analyses were also examined. Results: The survey-weighted prevalence of depressive symptoms was 6.9%, while that of poor self-rated health was 22.5%. Compared with contact four or more times per week, family or relative contact less than once per month was associated with higher odds of depressive symptoms (adjusted odds ratio [aOR] = 1.50, 95% confidence interval [CI]: 1.31–1.72) and poor self-rated health (aOR = 1.28, 95% CI: 1.17–1.41). Infrequent friend contact showed stronger associations with both outcomes (aOR = 2.02 and 1.77, respectively). Perceived neighborhood trust was associated with both outcomes, with significant age-related differences. Sensitivity analyses generally supported the principal findings. Conclusions: Distinct social relationship indicators were associated with depressive symptoms and self-rated health among adults living alone. These cross-sectional findings highlight the importance of differentiating interpersonal contact domains and neighborhood trust without implying causal effects.

## 1. Introduction

The rapid increase in the number of people living alone represents a major demographic and social transition with important implications for public health. Changes in family structure, population aging, delayed marriage, divorce, and greater diversity in living arrangements have contributed to the expansion of single-person households in South Korea [1,2]. Although living alone does not necessarily imply social isolation or poor health, individuals living alone may have fewer opportunities for routine interpersonal interaction within the household and may depend more heavily on relationships with family members, relatives, friends, and neighbors for emotional and practical support [3,4,5]. Consequently, understanding the health of adults living alone requires attention not only to household composition but also to the nature and frequency of social relationships maintained outside the household. Importantly, the social circumstances of individuals living alone may vary according to age, socioeconomic position, and access to interpersonal and community resources.

Living alone has been associated with mental health disparities in previous Korean studies. Population-based research has reported a higher prevalence of depression among individuals living alone than among those living with a spouse or other household members [6]. Research focusing specifically on Korean single-person households has also demonstrated an association between social isolation and depression and suggested that the magnitude of this association may differ across age groups [7]. More recent longitudinal evidence has further emphasized that single-person households are heterogeneous and that psychosocial and socioeconomic deprivation may be associated with different trajectories of depressive symptoms across the life course [8]. These findings suggest that living alone should not be treated as a uniform indicator of psychosocial vulnerability and that the specific social circumstances associated with mental health warrant further investigation.

Importantly, living alone, social isolation, social contact frequency, and social support are conceptually related but distinct constructs [3,9]. Living alone refers to a household arrangement, whereas social isolation generally concerns the extent to which individuals have limited social relationships or connections. Social contact frequency reflects the reported regularity of interpersonal contact and represents one observable aspect of social connectedness. In contrast, social support concerns the emotional, informational, or practical resources available through relationships and cannot be inferred directly from contact frequency. An individual may live alone while maintaining meaningful and supportive relationships despite relatively infrequent contact, whereas frequent interpersonal interaction does not necessarily indicate emotional closeness or adequate support. Previous research has linked structural and functional dimensions of social relationships to physical and mental health through several potential pathways, including emotional support, access to resources, behavioral regulation, and physiological processes [4,5,10,11,12]. Korean studies using Community Health Survey data have reported associations between contact with relatives, neighbors, and friends and depressive symptoms among older adults [13]. More recent research using the Korea Community Health Survey (KCHS) has also examined the moderating role of social networks in the association between living alone and depressive symptoms among older Korean adults [14]. However, measures of contact frequency should not be interpreted as direct indicators of loneliness, relationship quality, or the availability of social support.

Social relationships also extend beyond direct interpersonal contact. Neighborhood trust and reciprocity represent broader dimensions of social connectedness and social capital [15,16]. Unlike interpersonal contact frequency, which reflects the regularity of interaction with specific relationship groups, neighborhood trust concerns an individual’s perception of the trustworthiness of people in the surrounding community. Social capital has been associated with mental health in previous research, although associations may differ according to the dimensions of social capital examined [17,18]. Korean research using Community Health Survey data has similarly demonstrated associations between perceived neighborhood environments, including neighborhood trust, and depressive mood [19]. These findings suggest that interpersonal contact and neighborhood trust may capture distinct aspects of the social environment. Accordingly, they should be examined as separate indicators rather than combined indiscriminately into a single measure of social connectedness. Such conceptual differentiation is particularly relevant to adults living alone, whose interpersonal relationships and perceptions of their local communities may vary substantially.

Depressive symptoms are particularly relevant to the health of adults living alone. The Patient Health Questionnaire-9 (PHQ-9) is a widely used measure of depressive symptom severity [20] and has demonstrated acceptable reliability and validity in Korean populations [21,22]. The broader Patient Health Questionnaire was originally validated as a self-report version of the Primary Care Evaluation of Mental Disorders (PRIME-MD) [23]. A PHQ-9 score of 10 or higher is commonly used as a screening threshold for clinically relevant depressive symptoms [20,24]. However, this threshold identifies elevated depressive symptom levels rather than establishing a clinical diagnosis of depressive disorder. Previous Korean studies have identified socioeconomic disadvantage, health problems, limited social interaction, and insufficient social resources as factors associated with depressive symptoms among people living alone [7,8,14]. These findings indicate that depressive symptoms among adults living alone should be understood within a broader context encompassing individual characteristics, interpersonal relationships, and community resources. Examining distinct social relationship indicators may therefore contribute to a more differentiated understanding of the social circumstances associated with depressive symptoms.

Mental health, however, represents only one dimension of health among individuals living alone. Self-rated health (SRH) provides an individual’s overall subjective assessment of health and has been widely used as an indicator of general health status [25,26,27]. Previous research has demonstrated that self-rated health predicts subsequent mortality, including in studies accounting for selected objective health indicators [25,26]. Social relationships have also been linked to self-rated health [28]. Korean research has shown that individuals with smaller or more restricted social networks tend to report poorer self-rated health [29,30]. Among older adults living alone, social engagement has also been associated with self-rated health [31]. Although depressive symptoms and self-rated health are related, they represent distinct health outcomes. Depressive symptoms reflect psychological symptom burden, whereas self-rated health represents a broader subjective evaluation of overall health. Examining these outcomes within the same analytical framework may help identify whether different domains of social relationships demonstrate similar or distinct patterns of association with psychological and perceived general health.

Despite growing interest in single-person households, several gaps remain in the existing literature. First, previous Korean studies using Community Health Survey data have examined social isolation and depressive symptoms among adults living alone across age groups [7], as well as social contact and social network characteristics among older adults [13,14]. Building on this evidence, the present study examines family or relative contact, neighbor contact, friend contact, and perceived neighborhood trust as distinct indicators within a common survey-weighted analytical framework, while considering both depressive symptoms and poor self-rated health across three adult age groups. This approach enables comparisons across relationship domains and health outcomes without assuming that contact frequency, social isolation, social support, and neighborhood trust are interchangeable constructs. Second, previous studies have frequently examined depressive symptoms or self-rated health separately, whereas examining both outcomes within a common analytical framework may provide a more comprehensive understanding of their associations with social relationships. Studies of social network types among older Korean adults [29,32] offer relevant context, but their network-based measures are not directly comparable to the contact-frequency indicators used in the present study. Third, although social relationships are multidimensional, interpersonal contact with family members or relatives, neighbors, and friends, as well as perceived neighborhood trust, should not be regarded as interchangeable social resources. Their associations with health may differ by relationship domain and outcome [13,29,30]. Accordingly, further population-based research is warranted to examine these indicators separately within a common analytical framework while accounting for relevant sociodemographic characteristics.

To address these gaps, the present study used data from the 2025 Korea Community Health Survey (KCHS) [33,34] to examine associations between specific social relationship indicators, depressive symptoms, and self-rated health among adults living alone in South Korea. The study extends previous research by assessing three interpersonal contact domains—family members or relatives, neighbors, and friends—alongside perceived neighborhood trust and by comparing their associations with depressive symptoms and poor self-rated health across three adult age groups. Rather than treating living alone as equivalent to social isolation or interpreting contact frequency as a direct measure of social support, the study operationalized social relationships through separate indicators corresponding to the available survey measures.

Specifically, the objectives were to (1) describe the sociodemographic characteristics, social relationship indicators, depressive symptoms, and self-rated health of adults living alone; (2) examine associations between each social relationship indicator and the two health outcomes after adjustment for relevant sociodemographic characteristics; (3) investigate whether these associations differed across age groups; and (4) assess the robustness of the findings through sensitivity analyses incorporating continuous depressive symptom scores and additional health-related covariates. Because the study employed a cross-sectional design, all estimated associations were interpreted as observational relationships rather than evidence of causal effects.

## 2. Materials and Methods

### 2.1. Study Design and Data Source

This study employed a cross-sectional secondary data analysis using the 2025 Korea Community Health Survey (KCHS). The KCHS is a nationwide population-based health survey conducted annually by the Korea Disease Control and Prevention Agency (KDCA) in collaboration with local governments and public health centers. The survey is designed to produce standardized community-level health indicators among adults living in South Korea and collects comprehensive information on sociodemographic characteristics, health behaviors, physical and mental health, healthcare utilization, and social and environmental conditions.

The 2025 KCHS employed a complex multistage sampling design incorporating stratification, clustering, and sampling weights. Accordingly, the survey design characteristics must be considered when estimating population parameters and their corresponding standard errors. The original dataset used in the present study comprised 231,615 respondents. The analysis was restricted to adults living in one-person households, identified using the household-member count recorded in the survey dataset.

The primary explanatory variables comprised four social relationship indicators: frequency of contact with family members or relatives, frequency of contact with neighbors, frequency of contact with friends, and perceived neighborhood trust. The two primary health outcomes were depressive symptoms assessed using the Patient Health Questionnaire-9 (PHQ-9) and poor self-rated health. These outcomes were examined separately to determine whether different social relationship indicators exhibited distinct patterns of association with psychological and perceived general health.

Because the KCHS employs a complex sampling design, the analytical framework incorporated the survey design variables, including sampling strata, primary sampling units, and individual sampling weights, as described in Section 2.4. The application of the original survey weights was intended to account for the survey sampling design; however, weighting alone cannot eliminate potential selection bias arising from item nonresponse or the exclusion of participants with incomplete data. The overall study design and participant selection process are summarized in Figure 1.

### 2.2. Study Population

The original 2025 KCHS dataset included 231,615 respondents. Of these, 46,426 adults were identified as living in one-person households and were considered eligible for the present analysis. Participants were subsequently excluded according to the completeness and validity of the variables required for the primary analyses.

First, 121 participants with incomplete PHQ-9 data were excluded, leaving 46,305 individuals with complete depressive symptom data. Second, 1659 participants with incomplete information on one or more social relationship indicators were excluded, resulting in 44,646 participants. Third, one participant with missing self-rated health information was excluded, leaving 44,645 individuals. Finally, 477 participants with missing or invalid covariate data were excluded. The final analytic sample therefore comprised 44,168 adults living alone. The sequential participant selection process is illustrated in Figure 1.

A complete-case analytical approach was used, whereby participants were included only when valid information was available for the study outcomes, social relationship indicators, and covariates required for the primary regression models. This approach facilitated the use of a consistent analytical sample across the principal models. Nevertheless, complete-case analysis may introduce selection bias if the characteristics of excluded participants differ systematically from those of included participants.

Although the analyses incorporated the original KCHS sampling weights, these weights were not assumed to fully correct for potential bias associated with item nonresponse or complete-case selection. The implications of excluding participants with incomplete data are therefore considered when interpreting the findings and assessing their generalizability to adults living alone in South Korea. To further evaluate potential selection bias associated with complete-case analysis, the characteristics of participants included in the final analytic sample (*n* = 44,168) were compared with those excluded because of missing or invalid covariate information (*n* = 477). Comparisons were conducted among the 44,645 participants who had complete information on the primary health outcomes and social relationship indicators. Age, sex, age group, educational attainment, marital status, and economic activity were examined. Invalid or non-substantive responses were excluded separately for each variable. These comparisons were performed using unweighted descriptive statistics and conventional statistical tests as supplementary assessments of sample selection.

### 2.3. Measures

#### 2.3.1. Study Variables

Social relationships were assessed using four indicators included in the 2025 Korea Community Health Survey (KCHS): frequency of contact with family members or relatives, frequency of contact with neighbors, frequency of contact with friends, and perceived neighborhood trust. These indicators were examined separately because they represent distinct interpersonal and community-level dimensions of social connectedness.

The three contact-frequency indicators were derived from survey questions asking how often participants saw or contacted the family member or relative, neighbor, and friend with whom they had the most frequent contact. The original questionnaire distinguished contact with family members or relatives, contact with neighbors, and contact with friends other than neighbors. Importantly, the wording referred to seeing or contacting these individuals rather than exclusively to face-to-face encounters. Accordingly, the indicators were interpreted as reported frequency of social contact without assuming that all interactions occurred in person or through a particular communication medium.

Each contact-frequency variable comprised six response categories: less than once per month, once per month, two to three times per month, once per week, two to three times per week, and four or more times per week. These categories were retained in the primary analyses, with contact four or more times per week serving as the reference category. Higher and lower frequencies were interpreted as differences in the reported regularity of contact rather than as direct measures of relationship quality, emotional support, loneliness, or social isolation.

Perceived neighborhood trust was assessed using the KCHS item asking whether people in the respondent’s neighborhood could be trusted. Responses were classified as yes or no, with reported neighborhood trust serving as the reference category in the regression analyses. Unlike the three contact-frequency indicators, neighborhood trust reflects a perception of the local social environment rather than the frequency of interpersonal interaction.

The four indicators were entered simultaneously into the primary regression models to estimate their respective associations with depressive symptoms and self-rated health while accounting for the other social relationship indicators included in the model. The operational definitions and original questionnaire response categories are summarized in Supplementary Table S1.

#### 2.3.2. Depressive Symptoms

Depressive symptoms were measured using the Patient Health Questionnaire-9 (PHQ-9), included in the mental health section of the 2025 KCHS. The PHQ-9 comprises nine items assessing the frequency of depressive symptoms experienced during the preceding two weeks, including anhedonia, depressed mood, sleep disturbances, fatigue, appetite changes, negative self-perceptions, concentration difficulties, psychomotor changes, and thoughts of death or self-harm.

Each item is scored from 0 (not at all) to 3 (nearly every day), producing a total score ranging from 0 to 27, with higher scores indicating greater depressive symptom severity. Consistent with the established screening application of the PHQ-9, a total score of 10 or higher was used to identify participants with clinically relevant depressive symptoms, whereas scores below 10 were classified as not meeting this screening threshold [20,22,24]. This classification was not interpreted as a clinical diagnosis of depressive disorder.

The PHQ-9 total score was dichotomized for the primary analysis to facilitate the estimation and interpretation of odds ratios for clinically relevant depressive symptoms using complex-samples logistic regression. However, dichotomization may reduce information regarding variation in symptom severity and does not capture the full distribution of depressive symptoms. This limitation was considered when interpreting the findings. Participants with incomplete or invalid PHQ-9 responses were excluded from the primary analytical sample according to the study’s data-cleaning criteria.

#### 2.3.3. Self-Rated Health

Self-rated health (SRH) was measured using the general health assessment item included in the 2025 KCHS. Respondents rated their overall health using five response categories: very good, good, fair, poor, and very poor.

For the primary analysis, self-rated health was dichotomized into poor and non-poor self-rated health. Responses of poor or very poor were classified as poor self-rated health, whereas responses of very good, good, or fair were classified as non-poor self-rated health. This operationalization was used to distinguish respondents reporting an unfavorable overall health assessment from those reporting fair or better health.

Poor self-rated health was designated as the adverse outcome in the logistic regression models. Accordingly, odds ratios greater than 1.00 indicated higher odds of reporting poor self-rated health, whereas odds ratios below 1.00 indicated lower odds relative to the specified reference category. This interpretation was applied consistently across the analyses. Participants with missing or invalid responses to the self-rated health item were excluded from the analytical sample.

#### 2.3.4. Covariates

Covariates were selected based on their conceptual relevance to social relationships, depressive symptoms, and self-rated health and their availability in the 2025 Korea Community Health Survey (KCHS) dataset. The primary multivariable models included sex, age group, educational attainment, marital status, economic activity, annual household income, and average daily sleep duration.

Sex and age were included to account for demographic differences in interpersonal relationships, depressive symptoms, and perceived health. Age was categorized into three groups: 19–39 years, 40–64 years, and 65 years or older. Educational attainment was classified into eight categories: no formal education, Seodang/Han-hak, elementary school, middle school, high school, two- or three-year college, four-year university, and graduate school or higher. Marital status was categorized as married with spouse present, married with spouse absent, widowed, divorced, or never married. Economic activity was classified as yes or no.

Annual household income was calculated by converting reported monthly household income into an annual equivalent when necessary. Participants were classified into four income groups using the 25th, 50th, and 75th percentile cutoffs of the unweighted annual household income distribution. The resulting categories were Q1 (≤840), Q2 (>840 to ≤1680), Q3 (>1680 to ≤3600), and Q4 (>3600), expressed in the original survey income units. Participants with income values equal to a cutoff were assigned to the lower corresponding category; therefore, the four income groups did not contain equal numbers of respondents. Average daily sleep duration was calculated from reported weekday and weekend sleep durations using a weighted seven-day average and categorized as less than seven hours, seven to less than nine hours, and nine hours or more.

Sleep duration was included in the primary adjusted models because of its potential association with both social circumstances and mental or perceived health. Nevertheless, sleep duration may also be related to depressive symptom processes and could potentially lie along pathways connecting social relationships and health. Therefore, adjustment for sleep duration should not be interpreted as establishing a purely confounding role.

For additional sensitivity analyses, three health-related covariates were incorporated: self-reported physician diagnosis of hypertension, self-reported physician diagnosis of diabetes, and weekly walking frequency. Hypertension and diabetes were treated as categorical variables indicating reported diagnostic history, whereas walking frequency was modeled as a continuous variable representing the number of days walked during a typical week, ranging from zero to seven days. Invalid responses to the hypertension and diabetes items were treated as missing. These additional covariates were selected to assess whether the observed associations between social relationships and health outcomes persisted after accounting for selected chronic health conditions and physical activity.

The primary models adjusted for measured demographic and socioeconomic characteristics and average daily sleep duration, whereas the expanded sensitivity models additionally accounted for hypertension, diabetes, and weekly walking frequency. These analyses were conducted to evaluate the robustness of the observed associations across different model specifications rather than to establish causal relationships. Nevertheless, neither adjustment strategy can eliminate residual confounding. Unmeasured factors, including physical functional limitations, disability, and other health-related or social circumstances, may also influence the observed associations.

### 2.4. Statistical Analysis

All primary statistical analyses were conducted using IBM SPSS Statistics version 23.0 (IBM Corp., Armonk, NY, USA). The Complex Samples procedures were employed to account for the stratified and clustered sampling design of the 2025 Korea Community Health Survey (KCHS). The sampling design was specified using the stratification variable (KSTRATA), primary sampling unit identifier (SPOT_NO), and individual sampling weight (WT_P). These design variables were incorporated when estimating population characteristics, regression coefficients, and their corresponding standard errors.

Descriptive analyses were conducted to summarize the sociodemographic characteristics, social relationship indicators, depressive symptoms, and self-rated health of adults living alone. Unweighted frequencies and survey-weighted percentages with 95% confidence intervals (CIs) were reported. The survey-weighted prevalence of clinically relevant depressive symptoms, defined as a Patient Health Questionnaire-9 (PHQ-9) score of 10 or higher, and poor self-rated health was also estimated. Weighted prevalence estimates were additionally examined across three age groups: 19–39 years, 40–64 years, and 65 years or older.

Complex-samples logistic regression analyses were conducted separately for clinically relevant depressive symptoms and poor self-rated health. Both dependent variables were specified as binary outcomes, with elevated depressive symptoms and poor self-rated health representing the adverse outcome categories. Four social relationship indicators were entered simultaneously: frequency of contact with family members or relatives, frequency of contact with neighbors, frequency of contact with friends, and perceived neighborhood trust.

Two primary regression models were specified for each health outcome. Model 1 included all four social relationship indicators simultaneously without additional covariate adjustment. Thus, Model 1 estimates were mutually adjusted for the other social relationship indicators rather than completely unadjusted. Model 2 additionally adjusted for sex, age group, educational attainment, marital status, economic activity, annual household income group, and average daily sleep duration. The reference category for each contact-frequency variable was contact four or more times per week, whereas the reference category for neighborhood trust was a positive response indicating that people in the neighborhood could be trusted. Associations were reported as odds ratios (ORs) or adjusted odds ratios (aORs) with corresponding 95% CIs.

To examine potential age-related heterogeneity, interaction analyses were conducted using the fully adjusted complex-samples logistic regression framework. Separate interaction terms between age group and each of the four social relationship indicators were evaluated for both depressive symptoms and poor self-rated health, yielding eight interaction tests. The statistical significance of each interaction was assessed using design-adjusted Wald F tests. Given the number of interaction tests, a Bonferroni-adjusted significance threshold of 0.00625 (0.05/8) was considered when distinguishing more robust interaction findings from exploratory results.

Additional sensitivity analyses were conducted to evaluate the robustness of the principal findings. First, the PHQ-9 total score was analyzed as a continuous outcome using complex-samples general linear modeling, retaining the same social relationship indicators and adjustment covariates as in the primary fully adjusted model. Regression coefficients, standard errors, 95% CIs, and design-adjusted tests were examined to assess whether associations were consistent with those identified using the dichotomized PHQ-9 outcome.

Second, expanded complex-samples logistic regression models were fitted for both primary outcomes after additional adjustment for hypertension diagnosis, diabetes diagnosis, and weekly walking frequency. Hypertension and diabetes were entered as categorical covariates, whereas walking frequency was entered as a continuous covariate measured in days per week. Invalid diagnostic responses were excluded from these supplementary models. These analyses were intended to assess the robustness of associations to selected chronic health conditions and physical activity rather than to establish causal mechanisms.

Finally, to assess possible selection differences associated with the complete-case approach, characteristics of participants included in the final analytic sample were compared with those excluded because of missing or invalid covariate information. These supplementary comparisons were restricted to participants with complete primary outcome and social relationship information. Mean age was compared using an independent-samples *t*-test, whereas sex, age group, educational attainment, marital status, and economic activity were compared using Pearson’s chi-square tests. These comparisons used unweighted data and conventional statistical tests; therefore, their significance estimates were not interpreted as design-adjusted population-level inference. Invalid or non-substantive responses were excluded separately for each characteristic.

Statistical significance was evaluated using two-sided tests at an alpha level of 0.05 for the primary and sensitivity analyses, with the additional multiplicity consideration described above for age-related interactions. Category-specific regression estimates were distinguished from the overall significance of each categorical social relationship indicator. Because the data were cross-sectional, all associations were interpreted as observational rather than causal, and changes in estimated odds ratios after covariate adjustment were not regarded as direct evidence of specific confounding or mediating mechanisms.

### 2.5. Ethical Considerations

This study was conducted as a secondary analysis of de-identified data from the 2025 Korea Community Health Survey (KCHS), administered by the Korea Disease Control and Prevention Agency (KDCA). The dataset made available for research purposes did not contain directly identifiable personal information. The present study involved no direct interaction with participants, no additional data collection, and no intervention.

According to the KDCA, the KCHS has not been subject to Institutional Review Board (IRB) review since 2017 because the survey is not classified as human subjects research under the relevant provisions of the Enforcement Rule of the Bioethics and Safety Act. The present secondary analysis was conducted using the research dataset obtained through the official KCHS data access procedures. No additional informed consent was obtained from participants for this secondary analysis.

The analysis was limited to the available de-identified survey information, and the findings were reported in aggregate form to protect participant confidentiality.

## 3. Results

### 3.1. General Characteristics of the Study Population

The final analytical sample consisted of 44,168 adults living alone in South Korea. The sociodemographic characteristics of the study population are presented in Table 1 as unweighted frequencies and survey-weighted percentages with corresponding 95% confidence intervals (CIs).

After application of the survey weights, 49.7% of the participants were men and 50.3% were women. Regarding age distribution, 30.8% were aged 19–39 years, 34.2% were aged 40–64 years, and 34.9% were aged 65 years or older. These findings indicate that the analytical sample included adults across a broad age range, rather than being restricted to older individuals.

With respect to educational attainment, participants who had completed a four-year university education accounted for 29.0% of the weighted sample, followed by those with a high school education (27.3%). Regarding marital status, never-married participants represented 44.3% of the weighted sample, followed by widowed participants (26.0%) and divorced participants (18.3%). A total of 62.1% of participants reported engagement in economic activity.

In terms of annual household income, the third income quartile accounted for 37.8% of the weighted sample, followed by the highest income quartile (25.1%), the second quartile (21.0%), and the lowest quartile (16.1%). Regarding average daily sleep duration, 51.6% of participants reported sleeping less than seven hours, 45.1% reported sleeping seven to less than nine hours, and 3.3% reported sleeping nine hours or more per day.

Overall, the study population exhibited substantial demographic and socioeconomic diversity. This variation is relevant when interpreting the associations between social relationship indicators and health outcomes and provides a rationale for adjusting for sociodemographic characteristics in the multivariable analyses.

### 3.2. Distribution of Social Relationships, Depressive Symptoms, and Self-Rated Health

The distribution of social relationship indicators, depressive symptoms, and self-rated health among the 44,168 adults living alone is presented in Table 2. Unweighted frequencies and survey-weighted percentages with corresponding 95% confidence intervals (CIs) were used to describe the characteristics of the study population.

Regarding family or relative contact, 17.4% (95% CI: 16.9–17.9%) of the weighted population reported contact less than once per month, whereas 25.6% (95% CI: 25.1–26.1%) reported contact four or more times per week. Contact with neighbors was less frequent than contact with other relationship groups, with 55.8% (95% CI: 55.2–56.4%) reporting contact less than once per month and 18.0% (95% CI: 17.6–18.4%) reporting contact four or more times per week.

For contact with friends, 20.8% (95% CI: 20.4–21.3%) reported contact less than once per month, while 25.3% (95% CI: 24.7–25.8%) reported contact four or more times per week. These findings indicate that the frequency of interpersonal contact differed across family or relative, neighbor, and friend relationships.

With respect to perceived neighborhood trust, 56.0% (95% CI: 55.4–56.6%) of the weighted population reported that people in their neighborhood could be trusted, whereas 44.0% (95% CI: 43.4–44.6%) did not report neighborhood trust.

The survey-weighted prevalence of clinically relevant depressive symptoms, defined as a Patient Health Questionnaire-9 (PHQ-9) score of 10 or higher, was 6.9% (95% CI: 6.6–7.2%). The corresponding prevalence of poor self-rated health was 22.5% (95% CI: 22.0–23.0%). These findings demonstrate that depressive symptoms and unfavorable perceptions of general health were both present among adults living alone, although their estimated prevalence differed substantially.

Overall, the descriptive findings highlight considerable variation across social relationship domains and suggest that interpersonal contact frequency and neighborhood trust should be considered as distinct characteristics when examining their associations with psychological and perceived general health.

Additional survey-weighted descriptive analyses were conducted to examine the absolute prevalence of depressive symptoms and poor self-rated health across social relationship categories (Supplementary Table S3). The prevalence of depressive symptoms was 13.7% among participants reporting friend contact less than once per month, compared with 4.9% among those reporting contact four or more times per week. The corresponding prevalence of poor self-rated health was 41.8% and 17.5%, respectively. For family or relative contact, the prevalence of depressive symptoms was 12.2% among participants reporting contact less than once per month and 6.2% among those reporting contact four or more times per week. Notably, the prevalence of poor self-rated health was 18.0% among participants with neighbor contact less than once per month, compared with 33.5% among those reporting contact four or more times per week. Participants reporting neighborhood trust had a lower prevalence of depressive symptoms than those not reporting trust (5.1% versus 9.1%), whereas the corresponding prevalence of poor self-rated health was similar (22.7% versus 22.2%). These estimates are unadjusted survey-weighted descriptive findings and should be distinguished from the adjusted associations reported in the regression analyses.

### 3.3. Associations Between Social Relationships and Depressive Symptoms

The associations between social relationship indicators and clinically relevant depressive symptoms were examined using complex-samples logistic regression (Table 3). Model 1 included all four social relationship indicators simultaneously, whereas Model 2 additionally adjusted for sex, age group, educational attainment, marital status, economic activity, annual household income, and average daily sleep duration.

Design-adjusted omnibus Wald F tests for Model 2 revealed statistically significant overall associations with depressive symptoms for family or relative contact (F(5, 12,228) = 14.729, *p* < 0.001), neighbor contact (F(5, 12228) = 6.451, *p* < 0.001), friend contact (F(5, 12228) = 33.437, *p* < 0.001), and perceived neighborhood trust (F(1, 12232) = 88.322, *p* < 0.001). These tests assess the overall association of each social relationship indicator with depressive symptoms and complement the category-specific odds ratios presented in Table 3.

In Model 1, participants who reported contact with family members or relatives less than once per month had higher odds of clinically relevant depressive symptoms than those who reported contact four or more times per week (OR = 1.51, 95% CI: 1.33–1.72). Infrequent contact with friends showed a stronger association: participants reporting contact less than once per month had higher odds of depressive symptoms (OR = 2.63, 95% CI: 2.30–3.00). Participants who did not perceive their neighbors as trustworthy also had higher odds of depressive symptoms than those who reported neighborhood trust (OR = 1.68, 95% CI: 1.53–1.85).

Following adjustment for demographic, socioeconomic, and other prespecified covariates in Model 2, infrequent family or relative contact remained associated with higher odds of depressive symptoms (aOR = 1.50, 95% CI: 1.31–1.72). Contact with family members or relatives two to three times per week was associated with lower odds relative to contact four or more times per week (aOR = 0.80, 95% CI: 0.68–0.95). However, the associations across contact-frequency categories did not follow a consistent monotonic pattern.

For neighbor contact, participants reporting contact less than once per month had higher adjusted odds of depressive symptoms compared with those reporting contact four or more times per week (aOR = 1.45, 95% CI: 1.27–1.67). Contact once per week was also associated with higher adjusted odds (aOR = 1.27, 95% CI: 1.03–1.58). Nevertheless, the overall pattern did not demonstrate a uniform graded association across all categories.

Friend contact showed comparatively strong associations with depressive symptoms. Participants reporting contact with friends less than once per month had approximately twice the adjusted odds of clinically relevant depressive symptoms relative to those reporting contact four or more times per week (aOR = 2.02, 95% CI: 1.75–2.34). Contact once per month was also associated with higher adjusted odds (aOR = 1.29, 95% CI: 1.07–1.55), whereas several intermediate frequency categories had confidence intervals that included 1.00.

Perceived neighborhood trust was also associated with depressive symptoms. Participants who did not report neighborhood trust had higher adjusted odds of clinically relevant depressive symptoms than those reporting neighborhood trust (aOR = 1.62, 95% CI: 1.47–1.80).

Taken together, the results indicate that associations between social relationships and depressive symptoms differed across relationship domains and contact-frequency categories. Infrequent friend contact demonstrated a particularly strong association, while neighborhood trust was also independently associated with depressive symptoms after adjustment for measured covariates. These findings represent cross-sectional associations and should not be interpreted as evidence of causal effects.

### 3.4. Associations Between Social Relationships and Poor Self-Rated Health

The associations between social relationship indicators and poor self-rated health were examined using complex-samples logistic regression analyses (Table 4). Model 1 included all four social relationship indicators simultaneously, whereas Model 2 additionally adjusted for sex, age group, educational attainment, marital status, economic activity, annual household income, and average daily sleep duration.

In Model 1, participants reporting contact with family members or relatives less than once per month had higher odds of poor self-rated health than those reporting contact four or more times per week (OR = 1.40, 95% CI: 1.29–1.52). Infrequent friend contact showed a stronger association (OR = 3.47, 95% CI: 3.22–3.76). Participants who did not report neighborhood trust also had higher odds of poor self-rated health than those reporting neighborhood trust (OR = 1.21, 95% CI: 1.14–1.28).

In contrast, neighbor-contact frequency showed a different pattern in Model 1. Participants reporting neighbor contact less than once per month had lower odds of poor self-rated health compared with those reporting contact four or more times per week (OR = 0.37, 95% CI: 0.35–0.40). This association was substantially attenuated after adjustment for sociodemographic and other prespecified covariates.

In Model 2, infrequent family or relative contact remained associated with higher odds of poor self-rated health (aOR = 1.28, 95% CI: 1.17–1.41). The association for the lowest friend-contact category also remained statistically significant (aOR = 1.77, 95% CI: 1.63–1.93). Participants reporting friend contact once per month had higher adjusted odds of poor self-rated health than those reporting contact four or more times per week (aOR = 1.24, 95% CI: 1.11–1.39). However, the associations across intermediate contact-frequency categories did not demonstrate a consistent dose–response pattern.

For neighbor contact, the association with poor self-rated health changed substantially after covariate adjustment. In Model 1, participants reporting neighbor contact less than once per month had lower odds of poor self-rated health than those reporting contact four or more times per week (OR = 0.37, 95% CI: 0.35–0.40). However, this association was markedly attenuated in Model 2 (aOR = 0.95, 95% CI: 0.87–1.04). The adjusted odds ratios for the remaining neighbor-contact categories were also close to 1.00. These findings indicate that the associations observed in Model 1 were sensitive to covariate adjustment, although the analyses do not establish which specific covariates accounted for the attenuation.

Perceived neighborhood trust remained associated with poor self-rated health after adjustment for measured covariates. Participants who did not report neighborhood trust had higher adjusted odds of poor self-rated health than those who reported neighborhood trust (aOR = 1.36, 95% CI: 1.27–1.45).

Design-adjusted omnibus Wald F tests for Model 2 indicated statistically significant overall associations with poor self-rated health for family or relative contact (F(5, 12228) = 12.420, *p* < 0.001), friend contact (F(5, 12228) = 49.739, *p* < 0.001), and perceived neighborhood trust (F(1, 12232) = 77.940, *p* < 0.001). In contrast, the overall association for neighbor contact was not statistically significant (F(5, 12228) = 0.862, *p* = 0.505). These findings complement the category-specific adjusted odds ratios reported in Table 4.

Overall, the findings indicate that family or relative contact, friend contact, and neighborhood trust were associated with poor self-rated health after multivariable adjustment, whereas the initially strong association observed for neighbor-contact frequency was markedly attenuated. These results further illustrate that associations between social relationship indicators and health outcomes vary according to relationship domain and the covariates included in the analytical model.

### 3.5. Age-Related Differences in Social Relationships and Health Outcomes

Survey-weighted analyses revealed differences in the prevalence of depressive symptoms and poor self-rated health across age groups. The prevalence of clinically relevant depressive symptoms (PHQ-9 ≥ 10) was 5.6% among adults aged 19–39 years, 6.7% among those aged 40–64 years, and 8.1% among those aged 65 years or older. The corresponding prevalence of poor self-rated health was 5.7%, 18.0%, and 41.6%, respectively. These findings indicate that both adverse health outcomes were more prevalent in older age groups, with particularly pronounced age-related differences in poor self-rated health (Table 5).

To examine whether the associations between social relationships and health outcomes differed by age, separate interaction terms between age group and each of the four social relationship indicators were evaluated in fully adjusted complex-samples logistic regression models. A total of eight interaction tests were conducted across the two health outcomes.

For depressive symptoms, statistically significant interactions were observed between age group and family or relative contact (*p* = 0.040) and between age group and neighborhood trust (*p* = 0.003). Interactions involving neighbor contact (*p* = 0.228) and friend contact (*p* = 0.211) were not statistically significant.

For poor self-rated health, the interaction between age group and neighborhood trust was statistically significant (*p* < 0.001). The interaction involving neighbor contact was also significant at the conventional 0.05 level (*p* = 0.043), whereas those involving family or relative contact (*p* = 0.483) and friend contact (*p* = 0.917) were not statistically significant.

After applying a Bonferroni-adjusted significance threshold of 0.00625 to account for the eight interaction tests, only the age-group-by-neighborhood-trust interaction remained statistically significant for both depressive symptoms and poor self-rated health. The remaining interactions were therefore interpreted as exploratory findings.

To further characterize the significant age-group-by-neighborhood-trust interactions, age-specific adjusted odds ratios were estimated from the fully adjusted interaction models (Supplementary Table S2). For depressive symptoms, the inverse association with reported neighborhood trust was strongest among adults aged 19–39 years (aOR = 0.504, 95% CI: 0.398–0.637), followed by those aged 40–64 years (aOR = 0.568, 95% CI: 0.480–0.674) and those aged 65 years or older (aOR = 0.753, 95% CI: 0.653–0.868). A similar pattern was observed for poor self-rated health, with adjusted odds ratios of 0.470 (95% CI: 0.370–0.598), 0.736 (95% CI: 0.659–0.822), and 0.828 (95% CI: 0.759–0.905) across the three age groups, respectively. These findings indicate that the inverse associations between perceived neighborhood trust and adverse health outcomes were more pronounced among younger adults, although they do not establish causal effects.

These results suggest that the association between perceived neighborhood trust and health outcomes may vary across age groups among adults living alone. However, the findings should be interpreted as evidence of statistical interaction rather than causal age-dependent effects.

### 3.6. Sensitivity Analyses

Two supplementary sensitivity analyses were conducted to evaluate the robustness of the associations between social relationship indicators and health outcomes (Table 6).

First, the PHQ-9 total score was analyzed as a continuous outcome using complex-samples general linear modeling. The survey-weighted mean PHQ-9 score was 3.06, and the fully adjusted model explained approximately 11.6% of the variation in depressive symptom scores (R^2^ = 0.116). The overall associations were statistically significant for family or relative contact (Wald F = 24.677, *p* < 0.001), neighbor contact (Wald F = 13.909, *p* < 0.001), friend contact (Wald F = 60.387, *p* < 0.001), and neighborhood trust (Wald F = 220.445, *p* < 0.001).

Compared with participants reporting contact four or more times per week, those reporting family or relative contact less than once per month had higher PHQ-9 scores (B = 0.691, 95% CI: 0.534–0.848). Similarly, less frequent neighbor contact (B = 0.493, 95% CI: 0.373–0.613) and friend contact (B = 1.155, 95% CI: 1.010–1.300) were associated with higher depressive symptom scores. Participants reporting neighborhood trust had lower PHQ-9 scores than those who did not report neighborhood trust (B = −0.726, 95% CI: −0.822 to −0.630). These findings were broadly consistent with the primary analyses using the dichotomized PHQ-9 outcome.

Second, additional complex-samples logistic regression models were estimated after adjustment for hypertension diagnosis, diabetes diagnosis, and weekly walking frequency. These expanded models included 44,162 participants with valid data for all included variables.

In the expanded depressive symptoms model, infrequent family or relative contact remained associated with higher odds of clinically relevant depressive symptoms (aOR = 1.475, 95% CI: 1.283–1.695), as did infrequent neighbor contact (aOR = 1.409, 95% CI: 1.225–1.619) and infrequent friend contact (aOR = 1.910, 95% CI: 1.652–2.208). Participants reporting neighborhood trust had lower adjusted odds of depressive symptoms than those who did not report neighborhood trust (aOR = 0.624, 95% CI: 0.564–0.691).

For poor self-rated health, infrequent family or relative contact (aOR = 1.272, 95% CI: 1.154–1.402) and infrequent friend contact (aOR = 1.724, 95% CI: 1.578–1.883) remained associated with higher adjusted odds. Neighborhood trust was associated with lower odds of poor self-rated health (aOR = 0.746, 95% CI: 0.696–0.800). In contrast, the association between the lowest neighbor-contact category and poor self-rated health was not statistically significant (aOR = 0.958, 95% CI: 0.878–1.046).

In the expanded models, a higher number of walking days per week was associated with lower odds of depressive symptoms (aOR per additional day = 0.911, 95% CI: 0.895–0.927) and poor self-rated health (aOR = 0.909, 95% CI: 0.899–0.920). Hypertension and diabetes diagnosis indicators were also associated with both outcomes after adjustment for the other covariates.

Overall, these sensitivity analyses indicate that the principal associations involving family or relative contact, friend contact, and neighborhood trust were generally maintained when depressive symptoms were analyzed continuously and when selected chronic health conditions and walking frequency were incorporated into the adjusted models. These findings support the robustness of the observed associations to the alternative analytical specifications examined, without establishing causal relationships.

### 3.7. Comparison of Included and Excluded Participants

To assess potential selection differences associated with complete-case analysis, the characteristics of participants included in the final analytic sample (*n* = 44,168) were compared with those excluded because of missing or invalid covariate information (*n* = 477). These comparisons were restricted to the 44,645 potentially eligible participants with complete data on the primary health outcomes and social relationship indicators (Table 7).

The excluded and included groups did not differ significantly in mean age (62.77 ± 17.99 vs. 62.40 ± 18.58 years, respectively; *p* = 0.668), sex distribution (*p* = 0.183), or age-group distribution (*p* = 0.756). In contrast, statistically significant differences were observed in educational attainment (χ^2^ = 14.500, *p* = 0.043), marital status (χ^2^ = 11.126, *p* = 0.025), and economic activity status (χ^2^ = 9.646, *p* = 0.002).

Among participants with valid educational attainment data, the proportion reporting elementary school education was 17.0% in the excluded group and 22.9% in the included group. For economic activity, 50.6% of excluded participants and 57.7% of included participants reported engagement in economic activity.

Although only 1.1% of the potentially eligible participants were excluded at this stage, the observed sociodemographic differences suggest that potential selection bias associated with complete-case analysis cannot be ruled out. These supplementary comparisons were unweighted and should not be interpreted as design-adjusted population-level estimates.

## 4. Discussion

This study examined the associations between four social relationship indicators, depressive symptoms, and poor self-rated health among 44,168 adults living alone in South Korea using data from the 2025 Korea Community Health Survey (KCHS). Three findings are particularly relevant to the interpretation of the results. First, infrequent contact with friends and family members or relatives was associated with higher adjusted odds of both clinically relevant depressive symptoms and poor self-rated health, with particularly strong associations observed for infrequent friend contact. Second, neighbor-contact frequency was associated with depressive symptoms, whereas its association with poor self-rated health was substantially attenuated after adjustment for demographic, socioeconomic, and health-related covariates. Third, perceived neighborhood trust was associated with both health outcomes, and these associations varied significantly across age groups.

Together, these findings highlight the importance of distinguishing interpersonal contact domains from perceived neighborhood trust when examining health-related associations among adults living alone.

The associations between social relationships and depressive symptoms are broadly consistent with previous research identifying social relationships as important correlates of mental health [3,4,5,9,12,35,36]. Social ties may be associated with opportunities for interpersonal communication, access to resources, and perceptions of belonging [4,5,37,38]. Nevertheless, these potential pathways should be distinguished from the measures used in the present study. The KCHS contact-frequency questions assessed how often respondents saw or contacted particular relationship groups; they did not directly measure emotional support, relationship satisfaction, loneliness, or the quality of interpersonal interactions. Consequently, the findings identify associations involving reported contact frequency rather than demonstrating the psychological mechanisms through which social relationships may be related to health.

The present results also extend previous Korean population-based research. Yang et al. [13], using KCHS data from adults aged 65 years or older, identified associations between social contact and depressive symptoms, including the relevance of neighbor contact. Hwang and Ki [7] examined social isolation and depression among Korean adults living alone, while subsequent research highlighted heterogeneity in depressive symptom trajectories associated with psychosocial and socioeconomic circumstances [8]. Building on this evidence, the present study simultaneously examined contact with family members or relatives, neighbors, and friends, together with perceived neighborhood trust, across two health outcomes and three adult age groups. This approach provides a more differentiated assessment of associations among adults living alone without assuming that household composition, infrequent contact, and social isolation are equivalent.

A notable finding was the relatively strong association between infrequent friend contact and both adverse outcomes. Compared with participants who reported contacting friends four or more times per week, those reporting contact less than once per month had higher adjusted odds of clinically relevant depressive symptoms (aOR = 2.02, 95% CI: 1.75–2.34) and poor self-rated health (aOR = 1.77, 95% CI: 1.63–1.93). Previous research has indicated that social network composition and restricted social ties may be associated with differences in subjective physical and mental health [29,34]. Sohn et al. [29], in particular, identified variations in subjective health across social network types among older Korean adults. The present findings suggest that friend-contact frequency is a relevant relationship indicator in a broader adult population living alone.

One possible interpretation is that friendships may offer opportunities for companionship, shared activities, and voluntary social engagement. However, the present data cannot establish whether participants with frequent friend contact experienced greater emotional intimacy, stronger perceived support, or more satisfying relationships. Moreover, the associations were not uniformly graded across all six contact-frequency categories. Therefore, the results should not be interpreted as demonstrating that each incremental increase in friend-contact frequency produces a proportional improvement in health. Reverse causation is also plausible: depressive symptoms, physical limitations, or unfavorable perceptions of health may reduce opportunities or motivation to maintain interpersonal contact. Longitudinal research incorporating relationship quality and functional support measures is needed to clarify these possibilities.

Family or relative contact was also associated with both outcomes. Participants reporting contact less than once per month had higher adjusted odds of depressive symptoms (aOR = 1.50, 95% CI: 1.31–1.72) and poor self-rated health (aOR = 1.28, 95% CI: 1.17–1.41) than those reporting contact four or more times per week. Family relationships may represent potential sources of practical assistance and emotional connection for individuals without other household members [3,4,5]. Nevertheless, contact with family members should not automatically be equated with beneficial support. Family interactions may differ in emotional quality, perceived obligation, conflict, and personal expectations. The absence of a consistent dose–response pattern across contact-frequency categories further indicates that frequency alone cannot adequately characterize the health relevance of family relationships. These findings should therefore be interpreted as associations with reported contact frequency rather than evidence of the effects of supportive family functioning.

The findings concerning neighbor contact require particular caution. In the mutually adjusted model without additional sociodemographic covariates, participants reporting neighbor contact less than once per month had substantially lower odds of poor self-rated health than those reporting contact four or more times per week (OR = 0.37, 95% CI: 0.35–0.40). After adjustment for demographic, socioeconomic, and sleep-related covariates, this association was markedly attenuated (aOR = 0.95, 95% CI: 0.87–1.04). The direction of the initial estimate should not be interpreted as evidence that less frequent neighbor contact improves health. Rather, the substantial difference between models indicates that the estimated association is sensitive to the included covariates. Differences in age, socioeconomic circumstances, health status, and opportunities for neighborhood interaction may contribute to this pattern, although the present analyses cannot identify which factors account for the attenuation.

Neighbor contact showed a different pattern for depressive symptoms. Participants reporting contact with neighbors less than once per month had higher adjusted odds of depressive symptoms than those reporting contact four or more times per week (aOR = 1.45, 95% CI: 1.27–1.67). This finding is broadly compatible with previous evidence concerning neighbor contact and depressive symptoms among older Korean adults [13]. Nevertheless, the distinction between depressive symptoms and poor self-rated health should not be attributed to specific psychological or physiological mechanisms that were not assessed. Self-rated health encompasses a broader subjective evaluation of general health, potentially reflecting chronic conditions, functional limitations, and psychological experiences [25,26,27]. The present findings demonstrate outcome-specific patterns but do not establish why these patterns differ.

Perceived neighborhood trust demonstrated associations with both health outcomes after adjustment for contact frequency and the measured covariates. Participants who did not report neighborhood trust had higher adjusted odds of depressive symptoms (aOR = 1.62, 95% CI: 1.47–1.80) and poor self-rated health (aOR = 1.36, 95% CI: 1.27–1.45) than those reporting trust. Neighborhood trust reflects perceptions of the surrounding social environment and is conceptually distinct from the frequency of direct interpersonal contact. Previous studies have linked dimensions of social capital and perceived neighborhood environments with mental and general health [17,18,19,20,39]. Although these studies provide relevant conceptual and empirical context, their measures of social capital and neighborhood conditions are not necessarily equivalent to the neighborhood trust item used in the present analysis. The present findings are consistent with the possibility that perceptions of neighborhood trust capture contextual aspects of social connectedness not fully represented by contact-frequency measures. However, the analysis does not establish that increasing neighborhood trust would cause improvements in health.

The age-related interaction analyses provide additional evidence of heterogeneity. After applying the Bonferroni-adjusted significance threshold of 0.00625 across eight tests, the interaction between age group and neighborhood trust remained statistically significant for depressive symptoms (Wald F = 5.744, *p* = 0.003) and poor self-rated health (Wald F = 9.790, *p* < 0.001). In the fully adjusted interaction models, the inverse association between reporting neighborhood trust and depressive symptoms was more pronounced among adults aged 19–39 years (aOR = 0.504, 95% CI: 0.398–0.637) and 40–64 years (aOR = 0.568, 95% CI: 0.480–0.674) than among adults aged 65 years or older (aOR = 0.753, 95% CI: 0.653–0.868). For poor self-rated health, the corresponding estimates were 0.470 (95% CI: 0.370–0.598), 0.736 (95% CI: 0.659–0.822), and 0.828 (95% CI: 0.759–0.905), respectively (Supplementary Table S2).

These findings indicate that the associations between neighborhood trust and adverse health outcomes were not uniform across age groups. The comparatively stronger inverse associations among younger adults may reflect differences in social circumstances or the meaning of neighborhood trust across the adult life course. Such interpretations remain tentative because the study did not directly measure the processes underlying the observed age differences. In addition, the significant overall interaction tests do not establish that every pairwise difference between age groups was statistically significant. Other interactions that met the conventional 0.05 threshold, including family contact for depressive symptoms and neighbor contact for poor self-rated health, did not survive the Bonferroni adjustment and should be interpreted as exploratory.

The sensitivity analyses provided additional support for the consistency of the main findings across selected model specifications. When depressive symptoms were examined as a continuous PHQ-9 score, infrequent contact with family members or relatives and friends remained associated with higher symptom scores, while reported neighborhood trust was associated with lower scores. The broadly similar directions observed for the binary and continuous depressive symptom outcomes reduce concern that the principal findings were solely a consequence of the PHQ-9 dichotomization. Furthermore, expanded logistic regression models incorporating hypertension diagnosis, diabetes diagnosis, and weekly walking frequency yielded patterns generally consistent with the primary models. Nevertheless, these analyses do not eliminate the possibility of residual confounding, measurement error, or reverse causation. The additional health variables also do not comprehensively represent physical functioning or the severity of chronic illness.

The public health relevance of these associations should be interpreted in light of the observed prevalence of depressive symptoms and poor self-rated health, rather than solely on the magnitude of the adjusted odds ratios. The survey-weighted prevalence was 6.9% for clinically relevant depressive symptoms and 22.5% for poor self-rated health. These prevalence estimates provide population-level context for the adjusted odds ratios, but odds ratios should not be interpreted as risk ratios or as absolute differences in outcome probability. In particular, the higher prevalence of poor self-rated health indicates that relative odds may differ meaningfully from relative probabilities. Although the findings identify potentially relevant social relationship indicators, they do not quantify the absolute health benefits that would follow from changing contact frequency or neighborhood trust.

The study has several strengths. It used a large nationwide dataset and incorporated the KCHS stratification, clustering, and sampling weights. The analysis distinguished four social relationship indicators, examined two separate health outcomes, and included younger, middle-aged, and older adults living alone. The analysis also incorporated interaction testing, sensitivity analyses, and an examination of differences between included participants and those excluded at the final covariate-completeness stage. Together, these features strengthen the transparency of the findings and contribute to the literature by identifying differences across relationship domains, health outcomes, and age groups.

Several limitations warrant consideration. First, the cross-sectional study design precludes establishing temporal order or causal relationships. Poor mental or general health may affect interpersonal contact and perceptions of neighborhood trust, while unmeasured characteristics may influence both social relationships and health. Second, the primary social relationship variables were self-reported and assessed contact frequency or perceived neighborhood trust rather than relationship quality, functional support, loneliness, or objective network structure. Third, the PHQ-9 threshold identifies clinically relevant symptoms for screening purposes and does not establish a clinical diagnosis of depressive disorder. Although the continuous-score sensitivity analysis addressed one aspect of outcome operationalization, it did not resolve all potential measurement limitations.

Fourth, residual confounding remains possible despite adjustment for demographic, socioeconomic, sleep-related, and selected health-related variables. Functional limitations, disability, severity of chronic conditions, and other unmeasured social or health characteristics may influence the observed associations. Sleep duration may also occupy a complex position in the relationship between social circumstances and psychological health, rather than functioning exclusively as a confounding variable.

Fifth, complete-case selection may have affected the representativeness of the final analytic sample. Participants were excluded sequentially because of incomplete PHQ-9 data, social relationship information, self-rated health data, and covariate information. Among participants with complete outcome and social relationship data, the 477 individuals excluded at the final covariate stage did not differ significantly from the included sample in age or sex, but differences were observed in educational attainment, marital status, and economic activity. These comparisons were unweighted and exploratory, and they did not assess all participants excluded during earlier stages. Although the original survey weights accounted for the specified KCHS sampling design, they were not specifically recalibrated to address exclusions arising from the present complete-case analysis. Therefore, potential selection bias cannot be ruled out.

Finally, findings from the Korean population living alone should not automatically be generalized to people living in other household arrangements or to populations with different social and healthcare contexts. The use of broad age categories may also obscure variation within life stages. Future studies using longitudinal data, more detailed measures of social relationship quality and neighborhood experiences, and appropriately designed subgroup comparisons could help determine the extent to which the observed patterns persist across populations and over time.

From a public health perspective, the findings suggest that assessments of social circumstances among adults living alone may benefit from considering multiple relationship domains rather than relying on household composition or a single measure of contact frequency. Friend contact, family or relative contact, neighborhood trust, and neighbor contact showed different patterns of association with health outcomes. Community-based approaches addressing opportunities for social participation and neighborhood connectedness may warrant further evaluation. However, the present study does not demonstrate that interventions increasing interpersonal contact or neighborhood trust would improve depressive symptoms or self-rated health. Such potential benefits require prospective and intervention-based investigation.

## 5. Conclusions

This study examined the associations between social relationships, depressive symptoms, and self-rated health among 44,168 adults living alone in South Korea using the 2025 Korea Community Health Survey. The findings demonstrated that different dimensions of social relationships were associated with psychological and perceived general health in distinct ways. Infrequent contact with friends showed particularly consistent associations with higher adjusted odds of depressive symptoms and poor self-rated health, while infrequent family or relative contact and lack of neighborhood trust were also associated with both adverse outcomes. In contrast, the association between neighbor contact and poor self-rated health was substantially attenuated after adjustment for sociodemographic and other measured covariates.

Age-related interaction analyses further indicated that the associations between perceived neighborhood trust and both health outcomes differed across age groups, with stronger inverse associations observed among younger adults. These findings suggest that the relevance of neighborhood trust to health may vary across the adult life course. Sensitivity analyses using continuous depressive symptom scores and additional adjustment for selected health-related covariates generally supported the robustness of the principal associations.

Taken together, the results underscore the importance of distinguishing contact with family members or relatives, neighbors, and friends from perceived neighborhood trust when examining associations with depressive symptoms and self-rated health among adults living alone. However, the cross-sectional design does not establish causal relationships, and the observed associations may be influenced by reverse causation, residual confounding, and limitations inherent in self-reported measures. From a public health perspective, these results support further investigation of multidimensional social relationship indicators in identifying potentially vulnerable populations. Longitudinal and intervention-based studies are needed to determine whether strategies addressing social connectedness and neighborhood environments can improve mental health and self-rated health among adults living alone.

## Figures and Tables

**Figure 1:**
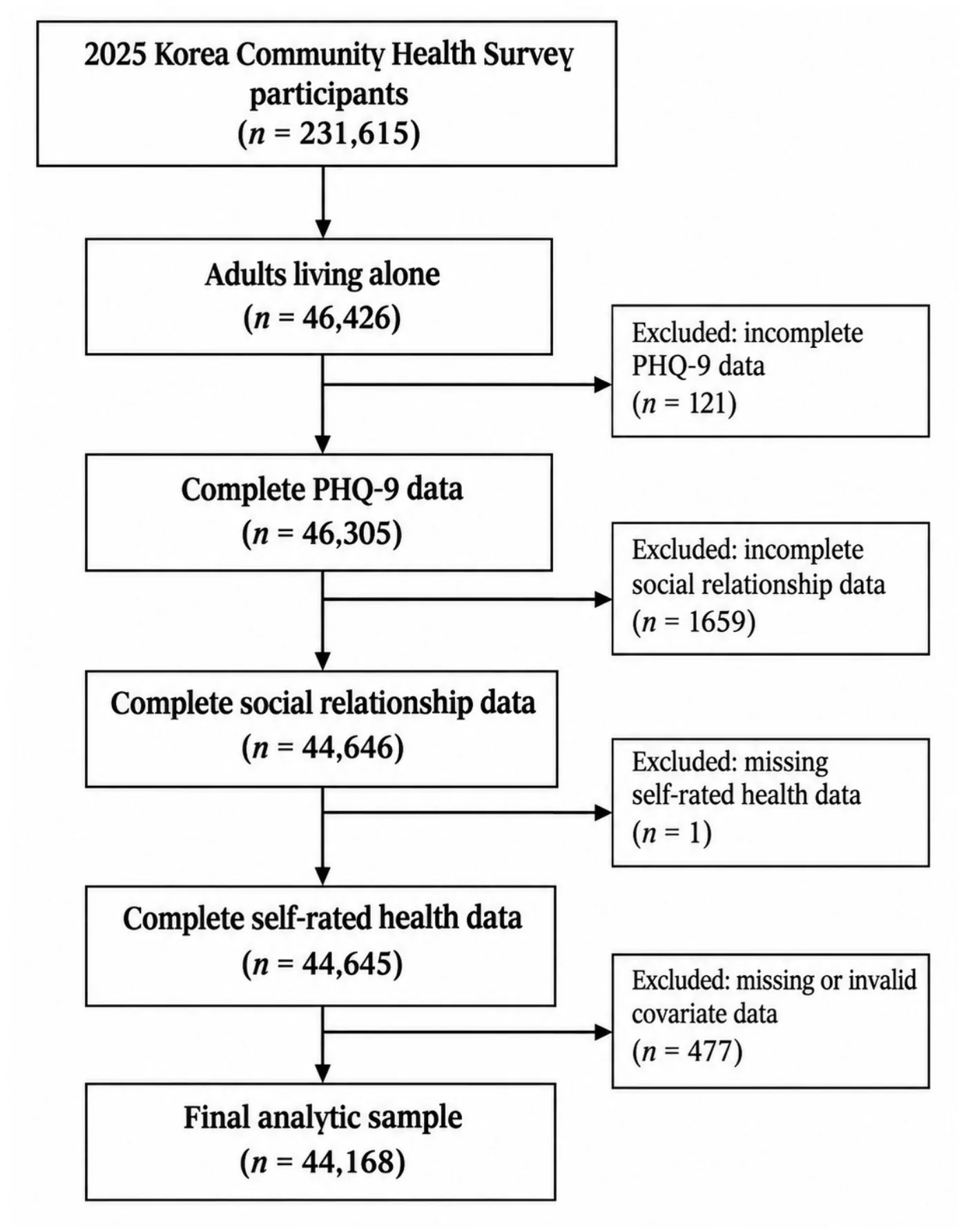
Flow diagram of participant selection for the final analytic sample.

**Table 1:** General Characteristics.

Characteristics	Category	Unweighted *n*	Weighted % (95% CI)
Sex	Male	18,205	49.7 (49.1–50.3)
	Female	25,963	50.3 (49.7–50.9)
Age group, years	19–39	7105	30.8 (30.1–31.5)
	40–64	13,577	34.2 (33.7–34.8)
	≥65	23,486	34.9 (34.4–35.5)
Education	No formal education	4723	4.7 (4.5–4.9)
	Seodang/Han-hak	61	0.1 (0.0–0.1)
	Elementary school	10,098	13.6 (13.2–13.9)
	Middle school	5212	9.7 (9.4–10.0)
	High school	10,958	27.3 (26.7–27.8)
	2-/3-year college	3482	11.1 (10.8–11.5)
	4-year university	8217	29.0 (28.4–29.6)
	Graduate school or higher	1417	4.5 (4.3–4.8)
Marital status	Married, spouse present	2361	5.1 (4.8–5.3)
	Married, spouse absent	3738	6.4 (6.1–6.6)
	Widowed	18,602	26.0 (25.5–26.5)
	Divorced	7687	18.3 (17.8–18.7)
	Never married	11,780	44.3 (43.6–44.9)
Economic activity	Yes	25,486	62.1 (61.5–62.7)
	No	18,682	37.9 (37.3–38.5)
Annual household income	Q1 (lowest)	11,254	16.1 (15.6–16.5)
	Q2	10,965	21.0 (20.5–21.4)
	Q3	14,107	37.8 (37.2–38.4)
	Q4 (highest)	7842	25.1 (24.6–25.7)
Average daily sleep duration	<7 h	23,468	51.6 (51.0–52.2)
	7–<9 h	19,011	45.1 (44.5–45.7)
	≥9 h	1689	3.3 (3.1–3.5)

**Table 2:** Distribution of Social Relationship Indicators, Depressive Symptoms, and Self-Rated Health Among Adults Living Alone (*n* = 44,168).

Variable	Category	Unweighted *n*	Weighted % (95% CI)
Family/relative contact	<1/month	6711	17.4 (16.9–17.9)
	1/month	4689	12.5 (12.1–12.9)
	2–3/month	5455	13.6 (13.2–14.1)
	1/week	5843	14.0 (13.6–14.4)
	2–3/week	7718	16.9 (16.5–17.4)
	≥4/week	13,752	25.6 (25.1–26.1)
Neighbor contact	<1/month	16,177	55.8 (55.2–56.4)
	1/month	2323	6.1 (5.8–6.4)
	2–3/month	2185	5.2 (4.9–5.4)
	1/week	2963	5.9 (5.6–6.1)
	2–3/week	5506	9.1 (8.8–9.5)
	≥4/week	15,014	18.0 (17.6–18.4)
Friend contact	<1/month	11,194	20.8 (20.4–21.3)
	1/month	4763	12.1 (11.7–12.5)
	2–3/month	4886	13.0 (12.6–13.4)
	1/week	4519	11.5 (11.1–11.9)
	2–3/week	7184	17.4 (16.9–17.8)
	≥4/week	11,622	25.3 (24.7–25.8)
Neighborhood trust	No	14,808	44.0 (43.4–44.6)
	Yes	29,360	56.0 (55.4–56.6)
Depressive symptoms	PHQ-9 < 10	41,234	93.1 (92.8–93.4)
	PHQ-9 ≥ 10	2934	6.9 (6.6–7.2)
Self-rated health	Non-poor	30,796	77.5 (77.0–78.0)
	Poor	13,372	22.5 (22.0–23.0)

Note: Values are presented as unweighted frequencies (*n*) and survey-weighted percentages with 95% confidence intervals (CIs). Social contact indicators represent the reported frequency of seeing or contacting family members or relatives, neighbors, and friends. Neighborhood trust reflects perceived trust in people living in the respondent’s neighborhood. Clinically relevant depressive symptoms were defined as a Patient Health Questionnaire-9 (PHQ-9) score ≥ 10. Poor self-rated health included responses of poor or very poor. Contact frequency should not be interpreted as a direct measure of relationship quality, social support, or loneliness.

**Table 3:** Multivariable Logistic Regression Analysis of the Association Between Social Relationships and Depressive Symptoms.

Social Relationship	Category	Model 1 OR (95% CI)	Model 2 aOR (95% CI)
Family/relative contact	<1/month	1.51 (1.33–1.72)	1.50 (1.31–1.72)
	1/month	0.86 (0.73–1.02)	0.96 (0.81–1.14)
	2–3/month	0.93 (0.79–1.10)	1.06 (0.89–1.25)
	1/week	0.83 (0.70–0.98)	0.90 (0.76–1.08)
	2–3/week	0.77 (0.65–0.90)	0.80 (0.68–0.95)
	≥4/week	1.00 (Reference)	1.00 (Reference)
Neighbor contact	<1/month	1.11 (0.97–1.26)	1.45 (1.27–1.67)
	1/month	1.02 (0.81–1.28)	1.22 (0.96–1.55)
	2–3/month	0.88 (0.69–1.13)	1.05 (0.82–1.36)
	1/week	1.10 (0.90–1.36)	1.27 (1.03–1.58)
	2–3/week	1.07 (0.90–1.28)	1.17 (0.98–1.40)
	≥4/week	1.00 (Reference)	1.00 (Reference)
Friend contact	<1/month	2.63 (2.30–3.00)	2.02 (1.75–2.34)
	1/month	1.22 (1.02–1.45)	1.29 (1.07–1.55)
	2–3/month	1.03 (0.85–1.25)	1.13 (0.92–1.38)
	1/week	0.95 (0.78–1.16)	1.03 (0.84–1.26)
	2–3/week	0.89 (0.74–1.06)	0.90 (0.75–1.09)
	≥4/week	1.00 (Reference)	1.00 (Reference)
Neighborhood trust	No	1.68 (1.53–1.85)	1.62 (1.47–1.80)
	Yes	1.00 (Reference)	1.00 (Reference)

Note: OR = odds ratio; aOR = adjusted odds ratio; CI = confidence interval. Model 1 included all four social relationship indicators simultaneously and therefore provided mutually adjusted associations among these indicators. Model 2 additionally adjusted for sex, age group, educational attainment, marital status, economic activity, annual household income, and average daily sleep duration. The reference categories were contact four or more times per week and reported neighborhood trust. Clinically relevant depressive symptoms were defined as PHQ-9 ≥ 10. Estimates were obtained using the complex survey design. Overall associations for each social relationship indicator were evaluated using design-adjusted omnibus Wald F tests in the fully adjusted model; the test statistics and p-values are reported in the Results section.

**Table 4:** Adjusted Associations Between Social Relationships and Poor Self-Rated Health.

Social Relationship	Category	Model 1 OR (95% CI)	Model 2 aOR (95% CI)
Family/relative contact	<1/month	1.40 (1.29–1.52)	1.28 (1.17–1.41)
	1/month	0.80 (0.73–0.89)	0.91 (0.81–1.02)
	2–3/month	0.86 (0.78–0.94)	1.01 (0.91–1.12)
	1/week	0.85 (0.77–0.93)	0.90 (0.82–1.00)
	2–3/week	0.85 (0.78–0.92)	0.91 (0.83–0.99)
	≥4/week	1.00 (Reference)	1.00 (Reference)
Neighbor contact	<1/month	0.37 (0.35–0.40)	0.95 (0.87–1.04)
	1/month	0.52 (0.45–0.59)	0.91 (0.79–1.05)
	2–3/month	0.54 (0.47–0.61)	0.91 (0.79–1.05)
	1/week	0.66 (0.59–0.74)	1.00 (0.88–1.14)
	2–3/week	0.78 (0.72–0.86)	1.01 (0.92–1.11)
	≥4/week	1.00 (Reference)	1.00 (Reference)
Friend contact	<1/month	3.47 (3.22–3.76)	1.77 (1.63–1.93)
	1/month	1.37 (1.24–1.52)	1.24 (1.11–1.39)
	2–3/month	1.08 (0.97–1.19)	1.09 (0.97–1.22)
	1/week	1.09 (0.98–1.21)	1.07 (0.95–1.20)
	2–3/week	1.03 (0.94–1.12)	0.97 (0.88–1.08)
	≥4/week	1.00 (Reference)	1.00 (Reference)
Neighborhood trust	No	1.21 (1.14–1.28)	1.36 (1.27–1.45)
	Yes	1.00 (Reference)	1.00 (Reference)

Note: OR = odds ratio; aOR = adjusted odds ratio; CI = confidence interval. Model 1 included all four social relationship indicators simultaneously, providing mutually adjusted estimates without additional sociodemographic or health-related covariates. Model 2 additionally adjusted for sex, age group, educational attainment, marital status, economic activity, annual household income, and average daily sleep duration. Contact four or more times per week and reported neighborhood trust were designated as reference categories. The outcome was poor self-rated health, defined as reporting poor or very poor overall health. An OR below 1.00 indicates lower odds of poor self-rated health relative to the reference category. All estimates were based on the complex survey analytical framework. Overall associations for each social relationship indicator were evaluated using design-adjusted omnibus Wald F tests in the fully adjusted model; the test statistics and *p*-values are reported in the Results section.

**Table 5:** Age-Group Interactions in the Associations Between Social Relationships and Health Outcomes (*n* = 44,168).

Social Relationship Indicator × Age Group	Depressive Symptoms Wald F	*p*	Poor Self-Rated Health Wald F	*p*
Family/relative contact	1.902	0.040	0.953	0.483
Neighbor contact	1.293	0.228	1.879	0.043
Friend contact	1.324	0.211	0.458	0.917
Neighborhood trust	5.744	0.003	9.790	<0.001

Note: Interaction effects were evaluated using design-adjusted Wald F tests from eight separate complex-samples logistic regression models. Each model included age group, sex, educational attainment, marital status, economic activity, annual household income group, average daily sleep duration, all four social relationship indicators, and one age-group interaction term. The numerator and denominator degrees of freedom were 10 and 12,223, respectively, for the contact-frequency interactions and 2 and 12,231, respectively, for the neighborhood-trust interactions. A Bonferroni-adjusted significance threshold of *p* < 0.00625 was applied across the eight interaction tests.

**Table 6:** Sensitivity Analyses of Social Relationships and Health Outcomes.

Social Relationship Indicator	Continuous PHQ-9 B (95% CI)	Depressive Symptoms aOR (95% CI)	Poor SRH aOR (95% CI)
Family/relative contact	0.691 (0.534–0.848)	1.475 (1.283–1.695)	1.272 (1.154–1.402)
Neighbor contact	0.493 (0.373–0.613)	1.409 (1.225–1.619)	0.958 (0.878–1.046)
Friend contact	1.155 (1.010–1.300)	1.910 (1.652–2.208)	1.724 (1.578–1.883)
Neighborhood trust	−0.726 (−0.822 to −0.630)	0.624 (0.564–0.691)	0.746 (0.696–0.800)

Note: For contact-frequency indicators, estimates compare less than once per month with four or more times per week. For neighborhood trust, estimates compare trust with no trust. Continuous PHQ-9 models adjusted for the original Model 2 covariates; expanded logistic models additionally adjusted for hypertension, diabetes, and walking frequency. The expanded logistic models included 44,162 participants. Unlike Table 3 and Table 4, neighborhood trust estimates in Table 6 compare respondents reporting trust with those reporting no trust. Thus, odds ratios below 1.00 indicate lower odds among respondents reporting neighborhood trust.

**Table 7:** Sociodemographic Characteristics of Participants Included in and Excluded from the Final Analytic Sample.

Characteristic	Excluded (*n* = 477)	Included (*n* = 44,168)	*p*-Value
Age, mean (SD), years	62.77 (17.99)	62.40 (18.58)	0.668
Sex			0.183
Male	211 (44.2%)	18,205 (41.2%)	
Female	266 (55.8%)	25,963 (58.8%)	
Age group, years			0.756
19–39	71 (14.9%)	7105 (16.1%)	
40–64	151 (31.7%)	13,577 (30.7%)	
≥65	255 (53.5%)	23,486 (53.2%)	
Educational attainment			0.043
No formal education	52 (11.2%)	4723 (10.7%)	
Seodang/Han-hak	0 (0.0%)	61 (0.1%)	
Elementary school	79 (17.0%)	10,098 (22.9%)	
Middle school	56 (12.0%)	5212 (11.8%)	
High school	127 (27.3%)	10,958 (24.8%)	
2-/3-year college	39 (8.4%)	3482 (7.9%)	
4-year university	88 (18.9%)	8217 (18.6%)	
Graduate school or higher	24 (5.2%)	1417 (3.2%)	
Marital status			0.025
Married, spouse present	40 (8.5%)	2361 (5.3%)	
Married, spouse absent	31 (6.6%)	3738 (8.5%)	
Widowed	191 (40.6%)	18,602 (42.1%)	
Divorced	86 (18.3%)	7687 (17.4%)	
Never married	122 (26.0%)	11,780 (26.7%)	
Economic activity			0.002
Yes	241 (50.6%)	25,486 (57.7%)	
No	235 (49.4%)	18,682 (42.3%)	

Note: Values are unweighted *n* (%) unless otherwise indicated. The denominator for percentages excludes invalid responses separately for each variable: educational attainment (excluded *n* = 465), marital status (excluded *n* = 470), and economic activity (excluded *n* = 476). Mean age was compared using an independent-samples *t*-test, and categorical characteristics were compared using Pearson’s chi-square tests. The educational-attainment comparison included one cell with an expected count below five; its asymptotic *p*-value should be interpreted cautiously. All tests were unweighted.

## Data Availability

The data analyzed in this study were obtained from the Korea Community Health Survey conducted by the Korea Disease Control and Prevention Agency. The data are available through the official KCHS data access procedures, subject to the applicable data-use requirements (https://chs.kdca.go.kr/chs/rdr/rdrInfoView.do accessed on 28 October 2026) [32].

## Supplementary Materials

The following supporting information can be downloaded at: https://www.mdpi.com/article/10.3390/healthcare14213793/s1. Table S1: Operational Definitions and Response Categories of Social Relationship Indicators; Table S2: Age-Specific Adjusted Associations Between Neighborhood Trust and Health Outcomes; Table S3: Survey-Weighted Prevalence of Depressive Symptoms and Poor Self-Rated Health Across Social Relationship Categories.
