# Supplementary material for: Social Relationships, Depressive Symptoms, and Self-Rated Health Among Adults Living Alone in South Korea: An Analysis of the 2025 Korea Community Health Survey
Source: Healthcare (Basel). 2026 Nov 6;14(21):3793. doi: 10.3390/healthcare14213793 (PMC13763111; doi:10.3390/healthcare14213793)
Supplement: Supplementary file 1 [file healthcare-14-03793-s001.zip › healthcare-4641299-supplementary.pdf]

Supplementary Materials

Social Relationships, Depressive Symptoms, and Self-Rated Health Among Adults Living Alone in South Korea: An Analysis of the 2025 Korea Community Health Survey

Man-Soo Kim

Table S1. Operational Definitions and Response Categories of Social Relationship Indicators

Indicator	English translation of official 2025 KCHS question	Analytical response categories	Reference category
Family/relative contact	How often do you see or contact the relative (including family members) with whom you have the most contact?	<1/month; 1/month; 2–3/month; 1/week; 2–3/week; ≥4/week	≥4/week
Neighbor contact	How often do you see or contact the neighbor with whom you have the most contact?	<1/month; 1/month; 2–3/month; 1/week; 2–3/week; ≥4/week	≥4/week
Friend contact	How often do you see or contact the friend (excluding neighbors) with whom you have the most contact?	<1/month; 1/month; 2–3/month; 1/week; 2–3/week; ≥4/week	≥4/week
Neighborhood trust	People in our neighborhood can trust one another. (Yes/No.)	Yes; No	Yes

Note: The four item wordings are English translations of the official Korean questions appearing in the 2025 KCHS Nationwide Common Questionnaire, printed p. 23 (PDF p. 24), section "Social and Physical Environment." The three contact items ask how often respondents "see or contact" the person with whom they have the most contact; they do not specify that contact must be face to face or explicitly enumerate telephone, text, or online modes. Contact response options were: less than once per month, once per month, 2–3 times per month, once per week, 2–3 times per week, and four or more times per week. Neighborhood trust was answered Yes or No. The English wording is a translation, not an official published English questionnaire. Reference categories are analytical choices. These items do not directly measure perceived loneliness, relationship quality, or functional social support.

Table S2. Age-Specific Adjusted Associations Between Neighborhood Trust and Health Outcomes

Age group	Depressive symptoms, aOR (95% CI)	Poor self-rated health, aOR (95% CI)
19–39 years	0.504 (0.398–0.637)	0.470 (0.370–0.598)
40–64 years	0.568 (0.480–0.674)	0.736 (0.659–0.822)
≥65 years	0.753 (0.653–0.868)	0.828 (0.759–0.905)

Note: aOR = adjusted odds ratio; CI = confidence interval; PHQ-9 = Patient Health Questionnaire-9. Odds ratios compare respondents reporting neighborhood trust (Yes) with respondents not reporting neighborhood trust (No; reference). Estimates derive from the two fully adjusted complex-sample logistic regression models including the age-group × neighborhood-trust interaction; the same analytical sample (N = 44,168) and survey design were retained while rotating the age-group reference category to obtain the corresponding 95% CIs. The models included family/relative, neighbor, and friend contact frequency, neighborhood trust, age group, sex, educational attainment, marital status, economic activity, annual household income group, and average daily sleep duration. Depressive symptoms were defined as PHQ-9 ≥10; poor self-rated health comprised poor or very poor responses. The overall interaction tests were $F(2, 12{,}231) = 5.744$ ($p = 0.003$) for depressive symptoms and $F(2, 12{,}231) = 9.790$ ($p < 0.001$) for poor self-rated health. Age-specific ORs are conditional associations; no pairwise age-group significance tests are implied.

Source: 2025 Korea Community Health Survey; analyses reported in the accompanying manuscript.

Table S3. Survey-Weighted Prevalence of Depressive Symptoms and Poor Self-Rated Health Across Social Relationship Categories

Social relationship indicator	Category (unweighted n)	Depressive symptoms, % (95% CI)	Poor self-rated health, % (95% CI)

Family or relative contact	<1/month (6,711)	12.2 (11.3–13.2)	31.7 (30.4–33.1)
	1/month (4,689)	5.9 (5.2–6.7)	18.5 (17.3–19.8)
	2–3/month (5,455)	6.2 (5.4–7.0)	19.4 (18.2–20.6)
	1/week (5,843)	5.5 (4.8–6.3)	19.7 (18.6–20.9)
	2–3/week (7,718)	4.8 (4.2–5.4)	19.3 (18.4–20.3)
	≥4/week (13,752)	6.2 (5.7–6.8)	23.4 (22.5–24.2)
Neighbor contact	<1/month (16,177)	7.8 (7.4–8.2)	18.0 (17.4–18.6)
	1/month (2,323)	6.0 (5.0–7.1)	20.6 (18.8–22.5)
	2–3/month (2,185)	5.1 (4.1–6.4)	21.4 (19.5–23.5)
	1/week (2,963)	6.2 (5.3–7.4)	25.1 (23.3–27.0)
	2–3/week (5,506)	5.9 (5.2–6.8)	28.2 (26.8–29.7)
	≥4/week (15,014)	5.6 (5.1–6.2)	33.5 (32.5–34.6)
Friend contact	<1/month (11,194)	13.7 (12.9–14.5)	41.8 (40.6–43.0)
	1/month (4,763)	6.3 (5.5–7.2)	20.2 (18.9–21.5)
	2–3/month (4,886)	5.4 (4.6–6.2)	16.3 (15.2–17.5)
	1/week (4,519)	4.8 (4.1–5.6)	16.7 (15.5–17.9)
	2–3/week (7,184)	4.5 (3.9–5.1)	16.6 (15.6–17.6)
	≥4/week (11,622)	4.9 (4.5–5.5)	17.5 (16.7–18.3)
Neighborhood trust	Yes (29,360)	5.1 (4.8–5.5)	22.7 (22.1–23.3)
	No (14,808)	9.1 (8.6–9.6)	22.2 (21.5–22.9)
**Overall**	**All (44,168)**	**6.9 (6.6–7.2)**	**22.5 (22.0–23.0)**

Note: Percentages are unadjusted survey-weighted row percentages and 95% confidence intervals obtained using the SPSS Complex Samples Crosstabs procedure (CSTABULATE), incorporating the KCHS stratification, cluster, and sampling-weight design. Unweighted category sample sizes (n) are shown in parentheses. Depressive symptoms were defined as PHQ-9 ≥10; poor self-rated health was defined as poor or very poor. Estimates refer to outcome prevalence within each social relationship category, not the distribution of relationship categories or covariate-adjusted predicted probabilities. N = 44,168 for all analyses. Contact-frequency categories refer to seeing or contacting the identified family member/relative, neighbor, or friend. Differences between categories are descriptive and must not be interpreted as causal effects or as necessarily statistically significant.
